# Nebulized heparin for patients under mechanical ventilation: an individual patient data meta-analysis

**DOI:** 10.1186/s13613-016-0138-4

**Published:** 2016-04-16

**Authors:** Gerie J. Glas, Ary Serpa Neto, Janneke Horn, Amalia Cochran, Barry Dixon, Elamin M. Elamin, Iris Faraklas, Sharmila Dissanaike, Andrew C. Miller, Marcus J. Schultz

**Affiliations:** Laboratory of Experimental Intensive Care and Anesthesiology (L·E·I·C·A), Department of Intensive Care, Academic Medical Center, Meibergdreef 9, 1105 AZ Amsterdam, The Netherlands; Department of Critical Care Medicine, Hospital Israelita Albert Einstein, São Paulo, Brazil; Department of Critical Care Medicine, Faculdade de Medicina do ABC, Santo André, Brazil; Program of Post-Graduation, Research and Innovation, Faculdade de Medicina do ABC, Santo André, Brazil; Department of Surgery, University of Utah Health Sciences Center, Salt Lake City, UT USA; Department of Intensive Care, St. Vincent’s Hospital, Melbourne, Australia; Division of Pulmonary, Critical Care, and Sleep Medicine, Department of Internal Medicine, James A. Haley Veteran’s Hospital, University of South Florida, Tampa, FL USA; Department of Surgery, Texas Tech University Health Sciences Center, Lubbock, TX USA; Department of Critical Care Medicine, Clinical Center, National Institutes of Health, Bethesda, MD USA; Department of Emergency Medicine, West Virginia University, Morgantown, WV USA

**Keywords:** Anticoagulants, Administration, Inhalation, Mechanical ventilation, Humans, Heparin, Intensive care

## Abstract

Pulmonary coagulopathy is a characteristic feature of lung injury including ventilator-induced lung injury. The aim of this individual patient data meta-analysis is to assess the effects of nebulized anticoagulants on outcome of ventilated intensive care unit (ICU) patients. A systematic search of PubMed (1966–2014), Scopus, EMBASE, and Web of Science was conducted to identify relevant publications. Studies evaluating nebulization of anticoagulants in ventilated patients were screened for inclusion, and corresponding authors of included studies were contacted to provide individual patient data. The primary endpoint was the number of ventilator-free days and alive at day 28. Secondary endpoints included hospital mortality, ICU- and hospital-free days at day 28, and lung injury scores at day seven. We constructed a propensity score-matched cohort for comparisons between patients treated with nebulized anticoagulants and controls. Data from five studies (one randomized controlled trial, one open label study, and three studies using historical controls) were included in the meta-analysis, compassing 286 patients. In all studies unfractionated heparin was used as anticoagulant. The number of ventilator-free days and alive at day 28 was higher in patients treated with nebulized heparin compared to patients in the control group (14 [IQR 0–23] vs. 6 [IQR 0–22]), though the difference did not reach statistical significance (*P* = 0.459). The number of ICU-free days and alive at day 28 was significantly higher, and the lung injury scores at day seven were significantly lower in patients treated with nebulized heparin. In the propensity score-matched analysis, there were no differences in any of the endpoints. This individual patient data meta-analysis provides no convincing evidence for benefit of heparin nebulization in intubated and ventilated ICU patients. The small patient numbers and methodological shortcomings of included studies underline the need for high-quality well-powered randomized controlled trials.

**Electronic supplementary material:**

The online version of this article (doi:10.1186/s13613-016-0138-4) contains supplementary material, which is available to authorized users.

## Background

Pulmonary coagulopathy is a characteristic feature of various forms of lung injury, including acute respiratory distress syndrome (ARDS) [1–4], pneumonia [1, 5, 6], and inhalation trauma [7]. Recently, it was even demonstrated that mechanical ventilation has the potential to alter the pulmonary hemostatic balance [8], with remarkably similar changes in coagulation and fibrinolysis as found in ARDS, pneumonia, or inhalation trauma [1, 4, 7, 9].

Fibrin deposition and hyaline membrane formation are considered important early features in diffuse alveolar damage, the hallmark of ARDS [1, 10–12]. Pulmonary activation of coagulation is likely to be involved in containing inflammation or infection to the site of injury and may have evolved as a host-protective mechanism [13, 14]. However, these local hemostatic disturbances could also be deleterious, as excessive or persistent fibrin deposition has been associated with alveolar collapse due to impaired surfactant function [15], pulmonary edema and impaired gas exchange [16], and eventually pulmonary fibrosis [17].

While preclinical studies provided support for the use of nebulized or systemic anticoagulants to prevent lung injury in animals [18, 19], clinical studies in ventilated patients thus far showed conflicting results [19, 20]. Clinical trials have been performed in patients with (mild) ARDS or sepsis, focusing on *systemic* treatment with anticoagulants such as recombinant human (rh)-activated protein C, antithrombin, rh-tissue factor pathway inhibitor, and unfractionated heparin. All but one trial were unsuccessful in improving patient outcomes [21–32]. It has been suggested that higher concentrations of an anticoagulant in the pulmonary compartment may be necessary to affect pulmonary disturbances [19]. Thus, *local* administration of anticoagulants to the pulmonary compartment could be considered a more effective anticoagulant intervention.

Over the last decades, nebulized heparin has been safely administered in a number of pulmonary conditions [33–35]. Studies in healthy volunteers showed nebulized heparin to reach the lower respiratory tract [36], distribute uniformly in the lungs [36], and exert local anticoagulant effects [35]. In line herewith, nebulized heparin attenuated pulmonary coagulopathy in critically ill patients with acute lung injury [37]. Intrapulmonary administered heparin crosses the alveolar membrane into the circulation, being absorbed rapidly and released gradually into the blood [38]. Indeed, there is evidence of a dose-dependent effect of heparin nebulization on plasma levels of aPTT [35, 39], with a threshold dose of 150,000 IU of heparin resulting in a measurable increase in aPTT [35]. This effect on systemic coagulation does not seem to potentiate the risk of bleedings [39–41], suggesting heparin nebulizations to be safe. Nevertheless, data on the feasibility and safety of heparin nebulizations in ventilated patients are scarce [19], and there are very limited data on the use of nebulized anticoagulants in ventilated patients. A systematic review recently showed conflicting effects of nebulized anticoagulation in burn patients with inhalation injury, a patient population in which this intervention is frequently applied [20]. It remains unclear whether nebulized anticoagulation is beneficial for all ventilated intensive care unit (ICU) patients. We performed an individual patient data meta-analysis to determine the association between nebulized anticoagulants and outcomes of intubated and ventilated ICU patients to test the hypothesis that nebulization of anticoagulants improves outcome.

## Methods

### Systematic search

Publications were identified through a systematic search of PubMed (1966–2014), Scopus, EMBASE, and Web of Science. Search terms referred to the intervention (nebulized, vaporized, aerosolized) and anticoagulant agents (anticoagulants, anticoagulation, antithrombins, heparin), as well as conditions of the patient population (acute lung injury, ARDS, critical illness, burn, smoke, inhalation injury) and mechanical ventilation. Searches were not limited by date or language. The detailed search strategy is shown in Additional file 1: Appendix 1.

Titles and available abstracts of the articles identified were screened. Studies were eligible for inclusion if they evaluated nebulized or aerosolized anticoagulants, including heparins, heparinoids, antithrombins, and/or fibrinolytics, in ventilated ICU patients. There were no restrictions regarding age of patients. Case reports and ongoing studies were excluded. Retrieved articles were screened for pertinent information, and reference lists of eligible articles were screened for potentially important papers. Quality of evidence for randomized and nonrandomized studies were assessed with use of, respectively, the Cochrane Collaboration’s tool for assessing risk of bias [42] and the Newcastle Ottawa Scale [43], see Additional file 1: Appendix 5.

### Collection of individual patient data

The corresponding author of each included study was contacted and asked for individual patient data. This included demographic and baseline characteristics, dose and duration of nebulized anticoagulants, duration of ventilation, occurrence of pneumonia, length of stay in the ICU and hospital, and mortality. Ventilatory parameters and lung injury scores (LIS) [44] were collected up to 7 days from admission. Data were accepted in any kind of electronic format.

### Primary outcome

The primary outcome was the number of ventilator-free days and alive at day 28, defined as the number of days alive and without ventilation until day 28.

### Secondary outcomes

Secondary outcomes included mortality during hospital stay, ICU-free days at day 28, defined as the number alive and outside the ICU at day 28, and hospital-free days and alive at day 28, defined as the number of days alive and outside hospital at day 28. PaO_2_/FiO_2_ and LIS at day seven, calculated from the available data, and occurrence of pneumonia during hospital stay.

### Statistical analysis

Continuous variables were presented as median and interquartile range (median [IQR]). Binary and categorical variables were presented as frequencies and percentages [*n* (%)].

Patients were analyzed according to use or not of nebulized anticoagulants. Time-to-event was defined as time from the day of inclusion in the study to the event of interest. We used a Cox proportional-hazards regression model to examine simultaneous effects of multiple covariates on outcomes, censoring patient data at the time of death, or hospital discharge. In all models, the categorical variables were tested for trend with the nonuse of nebulized anticoagulants as reference. The proportional-hazards assumption was assessed plotting partial residuals against survival time. A test for interaction between pairs of variables in the final model was performed. The effect of each variable in these models was assessed with the use of the Wald test and described by the hazard ratio with 95 % confidence interval (CI). The initial model included age and baseline PaO_2_/FiO_2_. The final model was developed by dropping each variable in turn from the model and by conducting likelihood-ratio tests to compare the full and the nested models. We used a significance level of 0.05 as the cutoff to exclude a variable from the model. Finally, use of nebulized anticoagulants (no vs. yes) was added to the model. Kaplan–Meier curves and log-rank test were used to determine the univariate significance of the study variables.

A linear mixed model was used to analyze time-course variables. A repeated-measures generalized linear model (GLM) was used to assess the time interaction for ventilatory and oxygenation parameters during mechanical ventilation. The model includes two factors: (1) study group (fixed factor), each level of the study group factor had a different linear effect on the value of the dependent variable; (2) time as covariate, time was considered to be a random sample from a larger population of values, and the effect was not limited to the chosen times.

Subgroup analyses were used to assess the effect of tidal volume size in the following prespecified subgroups: (1) age (<18 vs. ≥18 years); (2) dose of nebulized anticoagulant (low dose, defined as 30,000 U/day versus high dose, defined as ≥60,000 U/day); and (3) patient population (burn vs. non-burn). Propensity scores were estimated for each patient with logistic regression using two clinically relevant baseline characteristics (age and baseline PaO_2_/FiO_2_). Propensity score matching is described in detail in the supplemental material (Appendix file 1: Appendix 4). We conducted a post hoc sensitivity analysis in the matched cohort, including age (<18 vs. ≥18 years), dose of nebulized anticoagulant (low dose, defined as 30,000 U/day vs. high dose, defined as 60,000 U/day or higher), patient population (burn vs. non-burn), and tidal volume size (low, defined as ≤560 ml vs. high, defined as >560 ml by using the median as a cutoff value). All analyses were conducted with Review Manager v.5.1.1 (The Nordic Cochrane Centre, The Cochrane Collaboration, Copenhagen, Denmark), SPSS v.20 (IBM Corporation, New York, USA), and R v.2.12.0 (R Foundation for Statistical Computing, Vienna, Austria). For all analyses two-sided *P* values <0.05 were considered significant.

## Results

### Systematic search

**Fig. 1:**
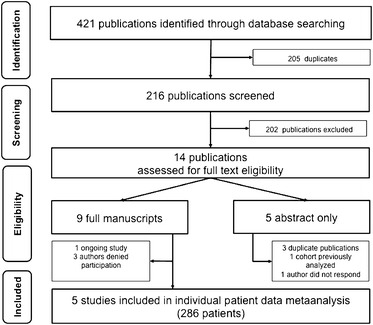
Prisma flow diagram showing the literature search and selection strategy

The search yielded 216 potentially relevant publications (Fig. 1). Based on the titles or abstracts, 202 publications were excluded. The remaining 14 publications reported on ten clinical studies, all on nebulized heparin [39–41, 45–55]. One publication reported on an ongoing trial [49]. Nine studies were eligible for inclusion in our individual patient data meta-analysis (521 patients). However, the corresponding authors of three studies did not provide the individual patient data [41, 45, 48], and one could not be contacted [53]. Therefore, data from five studies (286 patients) were available for the meta-analysis [39, 40, 50–52].

 Table 1 summarizes the study characteristics of the included studies. All three studies conducted in burn patients with inhalation injury were retrospective studies with historical controls [50–52]. One open label phase I study and one randomized controlled trial were conducted in critically ill patients [39, 40]. One study had a mixed population with both pediatric and adult patients [50], and all other studies were performed in adult patients. Dosage of heparin varied from 30,000 to 400,000 U/day.

**Table 1:** Characteristics of studies included in the individual patient data meta-analysis

Authors (year)	Design	Population (adult/pediatric)	Number of patients		Dose of heparin	Outcomes included in IPD meta-analysis	References
			Heparin	Control			
Holt (2008)	Retrospective with historical control	Smoke inhalation (adult and pediatric)	62	88	30,000	VFD-28; hospital mortality; pneumonia; PaO_2_/FiO_2_ at day 7; hospital-free days and alive at day 28	[50]
Dixon (2008)	Open label phase 1 trial	Critically ill (adult)	16	–	50,000–400,000	VFD-28; ICU mortality; ICU and hospital-free days and alive at day 28	[39]
Miller (2009)	Retrospective with historical control	Smoke inhalation (adult)	16	14	60,000	VFD-28; hospital mortality; PaO_2_/FiO_2_ and LIS at day 7; ICU and hospital-free days and alive at day 28; pneumonia	[52]
Dixon (2010)	Randomized controlled trial	Critically ill (adult)	25	25	150,000	VFD-28; hospital mortality; PaO_2_/FiO_2_ and LIS at day 7; ICU and hospital-free days and alive at day 28	[40]
Kashefi (2014)	Retrospective with historical control	Smoke inhalation (adult)	20	20	30,000	VFD-28; hospital mortality; pneumonia; PaO_2_/FiO_2_; and hospital-free days and alive at day 28	[51]

*VFD-28* ventilator-free days and alive at day 28, *IPD* individual patient data meta-analysis, *LIS* Lung injury scores

Of note, patients treated with nebulized heparin were ventilated with lower tidal volumes during the first 7 days of ventilation (Additional file 1: Appendix 3: Tables S1 and Appendix 4: Table S5). All other ventilatory parameters were similar between the two study groups (Additional file 1: Appendix 2: Figures S2 and S3; Appendix 3: Table S1).

Table 2 summarizes the demographic data of the included patients. For the propensity score-matched cohort, 248 patients could be analyzed (Additional file 1: Appendix 4: Table S3).

**Table 2:** Characteristics of the patients included in the individual patient data analysis

Variables	Overall cohort (*N* = 286)		
	Nebulized heparin (*N* = 139)	Control (*N* = 147)	SD (%), *P*
Age, years	50.0 (36.0–69.0)	45.0 (31.0–63.0)	17.6, 0.09
	(*N* = 139)	(*N* = 147)	
Gender, male (%)	81 (65.9)	107 (72.8)	−19.0, 0.14
APACHE III	22.0 (17.0–31.0)	24.0 (15.0–32.0)	5.1, 0.74
	(*N* = 57)	(*N* = 39)	
% TBSA	25.5 (12.9–52.2)	31.2 (16.5–52.2)	−5.1, 0.51
	(*N* = 90)	(*N* = 110)	
Dosage of heparin (U/day)	30,000	0.0	–
	(30,000–100,000)	(0.0–0.0)	
Dosage of NAC (mg/day)	3600 (3600–3600)	0.0 (0.0–0.0)	–
Duration of treatment	7.0 (3.0–12.0)	0.0 (0.0–0.0)	–
Baseline LIS	2.0 (0.7–2.5)	2.0 (1.2–3.0)	−26.2, 0.29
	(*N* = 41)	(*N* = 39)	
Baseline PaO_2_/FiO_2_	219.5 (158.2–316.5)	270.0 (163.5–366.5)	−18.3, 0.09
	(*N* = 136)	(*N* = 141)	

Values are median (IQR) or no./total no. (%). Not all requested data were available for each study

*SD* standardized difference, *TBSA* total burn surface area, *NAC* *N*-acetylcysteine, *LIS* lung injury scores, *N* number of patients

### Effects of heparin on outcome

The median number of ventilator-free days and alive at day 28 did not differ in patients treated with nebulized heparin compared to patients in the control group (14, IQR 0–23 vs. 6, IQR 0–22 days, *P* = 0.459). A statistically significant difference was found for ICU-free days at day 28 (3 [0–19] vs. 0 [0–14] days, *P* = 0.035). The LIS at day seven were also significantly lower in patients treated with nebulized heparin (2.0 [1.0–2.5] vs. 2.2 [1.7–3.0] days, *P* = 0.027). There was no difference in hospital mortality (Table 3 and Additional file 1: Appendix 2: Figure S1), hospital-free days and alive at day 28 or occurrence of pneumonia during hospital stay (Table 3).

**Table 3:** Primary and secondary outcomes

Variables	Nebulized heparin (*N* = 139)	Control (*N* = 147)	Odds ratio^a^ (95 % CI)	*P*
*Primary outcome*				
Ventilator-free days at day 28	14.0 (0.0–23.0)	6.0 (0.0–22.0)		0.459
	(*N* = 139)	(*N* = 144)		
*Secondary outcomes*				
Overall mortality	34/139 (24.5)	35/147 (23.8)	0.65 (0.50–1.56)^b^	0.653
	(*N* = 139)	(*N* = 147)		
PaO_2_/FiO_2_ at day seven (mmHg)	242.5 (206.0–300.0)	220.2 (179.4–297.7)		0.098
	(*N* = 61)	(*N* = 78)		
LIS at day seven	2.0 (1.0–2.5)	2.2 (1.7–3.0)		0.027
	(*N* = 40)	(*N* = 48)		
Pneumonia during hospital stay	48/82 (58.5)	48/106 (45.3)	1.49 (0.79–2.80)	0.219
	(*N* = 82)	(*N* = 106)		
ICU-free days at day 28	2.9 (0.0–19.0)	0.0 (0.0–14.2)		0.035
	(*N* = 78)	(*N* = 62)		
Hospital-free days at day 28	0.0 (0.0–12.0)	0.0 (0.0–14.0)		0.951
	(*N* = 139)	(*N* = 147)		

Values are median (IQR), and others are no./total no. (%)

Not all requested data were available for each study

*LIS* lung injury scores, *CI* confidence interval, *N* number of patients

^a^Adjusted by: age and baseline PaO_2_/FiO_2_

^b^Presented as hazard ratio adjusted by: age, %TBSA, and baseline PaO_2_/FiO_2_

In subgroup analyses, there was no difference in number of ventilator-free days at day 28, overall mortality nor number of hospital-free days and alive at day 28, according to age (<18 vs. ≥18 years), dose of heparin, type of population and tidal volume size (Additional file 1: Appendix 3: Table S2).

### Propensity score-matched cohort

 Results of the meta-analysis in the propensity score-matched cohort are presented in the online supplement (Additional file 1: Appendix 4: Tables S3–S6).

The median number of ventilator-free days at day 28 in patients treated with nebulized heparin was higher than that in control patients (16 [0–23] vs. 5 [0–20] days), but again this difference did not reach statistical significance (*P* = 0.133). Also, no statistical differences were found for the number of ICU-free days and alive at day 28 and LIS at day seven and other secondary endpoints (Additional file 1: Appendix 2: Figure S1 and Appendix 4: Table S4).

Also in this part of the analysis, it was found that patients treated with nebulized heparin were ventilated with lower tidal volumes than control patients during the first 7 days of ventilation (Additional file 1: Appendix 4: Table S5). In the post hoc sensitivity analysis on age, dose of heparin, type of population and tidal volume size, no differences were found for ventilator-free days and hospital-free days at day 28 (Additional file 1: Appendix 4: Table S6).

## Discussion

Nebulization of heparin, alone or combined with other agents, did not improve the outcome of mechanically ventilated patients in this individual patient data meta-analysis. Even though patients who received nebulization with heparin demonstrated higher numbers of ventilator-free days and alive at day 28, differences were not statistically significant. We did find a higher number of ICU-free days and alive at day 28 and lower LIS at day seven in patients treated with nebulized heparin. A propensity score-matched cohort analysis, however, showed no beneficial effects of heparin nebulization.

The aim of this individual patient data meta-analysis was to investigate the effectiveness of nebulized anticoagulants in intubated and ventilated ICU patients. Since heparin was the only anticoagulant agent used in the included studies, we are unable to ascertain the potential efficacy of any other anticoagulant, due to paucity of available evidence. Also, the majority of patients included were patients with inhalation injury (220 of 286). Thus, conclusions on the effects of nebulized heparin for intubated and ventilated ICU patients in general cannot be made. As adverse effects of mechanical ventilation may be more severe in burn patients, it is possible that these patients benefit more from nebulized anticoagulants compared to non-burn or smoke inhalation patients [19].

Reported effects of nebulized heparin on duration of mechanical ventilation and other outcomes such as mortality in patients with inhalation injury have been conflicting. Beneficial effects of heparin nebulization could have been confounded by improvements in ICU care in general as they were conducted around a change in institutional protocol [45, 52]. In two other before–after studies no beneficial effects of heparin nebulizations were seen [50, 51]. Furthermore, in three of the included studies [50–52], nebulized heparin was combined with the use of mucolytic agents and bronchodilators. This highlights the difficulty to distinguish between the effects of heparin nebulization and other parts of treatment on patient outcome in retrospective studies with historical controls.

One important finding of our individual patient data meta-analysis was that patients receiving heparin nebulization were ventilated with lower tidal volumes compared to control patients. While in theory improved clinical outcomes could have been caused by nebulization of heparin, it could also function as an important confounder, since low tidal volume ventilation is associated with a better outcome, also in patients without ARDS [56–60]. Still, relatively high tidal volumes were used in all included studies which may hamper extrapolation to current ventilation practices. On the other hand, while lower tidal volumes are increasingly being used [61, 62], guidelines inconsistently advise on tidal volume size in ICU patients without ARDS and current ventilation practice is uncertain [63].

Dosage of heparin varied from 30,000 to 400,000 U/day. Several studies suggested a dose-dependent effect of heparin nebulization in which dosages of 30,000 U/day improved outcomes in pediatric patients [45] but failed to improve outcomes in adults [50, 51], while higher dosages did improve outcome of adult patients [48, 52]. The present meta-analysis could not confirm this. Types of nebulizers and its position in the circuit may affect the delivery of nebulized drugs in ventilated patients [64–66]. Furthermore, aerosol particle size distribution and heparin concentrations may also influence the amount of heparin delivered to the lower respiratory tract [67]. The method of nebulization differed between studies. Three studies used mesh nebulizers [39, 40, 50], and two studies used jet nebulizers [51, 52]. Thus, the delivered amount of nebulized drugs may have varied.

Our results contradict the conclusion of a previous systematic review concluding that inhaled anticoagulation regimens improve survival and decrease morbidity in smoke inhalation patients [20]. This may be due to some major differences between the two studies. First, as our aim was to investigate the effect of heparin nebulization in any critically ill patient, we included different studies. Second, the use of individual patient data allowed standardization of the analyses across studies irrespective of how the data were reported [68].

One major limitation of this meta-analysis is that we were only able to analyze the individual data of 286 patients out of 521 potentially eligible patients as the authors of four studies did not provide individual patient data. Other limitations are caused by the methodological shortcomings of included studies. Only one of the included studies was a small, but properly conducted randomized controlled trial [40]. The other studies, mostly small in size, used an open label design or were retrospective cohort studies with use of historical controls. Due to these limitations, the results from this meta-analysis should be interpreted with great caution. To account for some of those limitations we used propensity score matching correcting for relevant baseline characteristics. However, imbalances such as the presence of unmeasured confounders are likely to remain [69]. Nevertheless, the post hoc sensitivity analysis indicates that the results of this meta-analysis were affected neither by factors such as age, presence of burn, or inhalation injury nor by differences in tidal volume size and heparin dosages.

## Conclusion

 No beneficial effects of heparin nebulization on the outcome of ventilated patients were observed in this individual patient data meta-analysis. The small patient numbers and methodological shortcomings of included studies underline the need for high-quality well-powered randomized controlled trials to determine the effect of heparin nebulization on outcome of intubated and ventilated ICU patients.

## Additional file

10.1186/s13613-016-0138-4 Online supplemental material.

## Abbreviations

ARDS: acute respiratory distress syndrome
CI: confidence interval
GLM: generalized linear model
GRADE: grading of recommendations assessment, development and evaluation
ICU: intensive care unit
IQR: interquartile range
LEICA: Laboratory of Experimental Intensive Care and Anesthesiology
LIS: lung injury scores
SD: standard deviation
