# Supplementary material for: Nebulized heparin for patients under mechanical ventilation: an individual patient data meta-analysis
Source: Ann Intensive Care. 2016 Apr 16;6:33. doi: 10.1186/s13613-016-0138-4 (PMC4833759; doi:10.1186/s13613-016-0138-4)
Supplement: Supplementary file 1 — 10.1186/s13613-016-0138-4 Online supplemental material. [file 13613_2016_138_MOESM1_ESM.doc]

**Online supplement to:**

**Nebulized Heparin for Patients Under Mechanical Ventilation: an Individual Patient Data Meta-analysis**

Gerie J. Glas MD,^1,2^ Ary Serpa Neto MD MSc PhD,^3,4,5^ Janneke Horn MD PhD,^1,2^ Amalia Cochran MD FACS,^6^ Barry Dixon MBBS PhD,^7^ Elamin M. Elamin MD MSc,^8^ Iris Faraklas BSN,^6^ Sharmila Dissanaike,^9^ Andrew C Miller MD,^10,11^ and Marcus J. Schultz MD PhD^1,2^

**Academic Medical Center; Amsterdam, The Netherlands**

^1^Department of Intensive Care

^2^Laboratory of Experimental Intensive Care and Anesthesiology (L·E·I·C·A)

**Hospital Israelita Albert Einstein; São Paulo, Brazil**

^3^Department of Critical Care Medicine

**Faculdade de Medicina do ABC; Santo André, Brazil**

^4^Department of Critical Care Medicine

^5^Program of Post-Graduation, Research and Innovation;

**University of Utah Health Sciences Center; Salt Lake City, Utah, USA**

^6^ Department of Surgery

**St. Vincent’s Hospital; Melbourne Australia**

^7^Department of Intensive Care

**James A. Haley Veteran’s Hospital and University of South Florida; Tampa, Florida, USA**

^8^Department of Internal Medicine, Division of Pulmonary, Critical Care, and Sleep Medicine

**Texas Tech University Health Sciences Center, Lubbock, Texas, USA**

^9^Department of Surgery

**National Institutes of Health; Bethesda, Maryland, USA**

^10^Department of Critical Care Medicine; Clinical Center;

**West Virginia University; Morgantown, West Virginia, USA**

^11^Department of Emergency Medicine

**Corresponding author**

Gerie J. Glas, MD

Department of Intensive Care & Laboratory of Experimental Intensive Care and Anesthesiology (L·E·I·C·A)

Academic Medical Center

Meibergdreef 9, 1105 AZ Amsterdam, The Netherlands

[g.j.glas@amc.uva.nl](mailto:g.j.glas@amc.uva.nl)

**List of Contents**

**Appendix 1: Search Strategy**

**Appendix 2: Figures – Ventilation data and subgroup analysis**

**Figure S1:** Kaplan-Meier Estimates of the Probability of the Secondary Outcome.

**Figure S2:** Ventilatory parameters in patients treated with nebulized heparin (*black dotted line*) and controls (*black solid line*).

**Figure S3:** Oxygenation parameters in patients treated with nebulized heparin (*black dotted line*) and controls (*black solid line*).

**Appendix 3: Tables – Ventilation data and subgroup analysis**

**Table S1:** Ventilation and oxygenation parameters in the overall cohort

**Table S2:** Outcomes of patients in different subgroups (nebulized heparin *vs.* control)

**Appendix 4: Propensity matched cohort**

**Table S3:** Characteristics of the patients included in the individual patient data analysis

**Table S4:** Primary and secondary outcomes

**Table S5:** Ventilation and oxygenation parameters

**Table S6:** Post-hoc sensitivity analysis in the matched cohort (control vs heparin)

**Appendix 5: Assessment of Quality of Evidence of included studies**

**APPENDIX 1 – Search Strategy**

Detailed search strategies of PubMed, Scopus, EMBASE, and Web of Science was to capture relevant published studies of nebulized anticoagulation regimens in mechanically ventilated patients.

**PubMed – 87 results**

(aerosols[mesh] OR nebulizers and vaporizers[mesh] OR administration, inhalation[mesh] OR nebulize[tiab] OR nebulized[tiab] OR nebulizer[tiab] OR nebulizers[tiab] OR nebulization[tiab] OR aerosol[tiab] OR aerosols[tiab] OR aerosolized[tiab] OR inhale[tiab] OR inhaled[tiab] OR inhalation[tiab] OR inhalable[tiab] OR vaporize[tiab] OR vaporizer[tiab] OR vaporizers[tiab]) AND (heparin[mesh] OR anticoagulants[mesh] OR antithrombins[mesh] OR heparin[tiab] OR anticoagulant[tiab] OR anticoagulants[tiab] OR anticoagulation[tiab] OR antithrombin[tiab] OR antithrombins[tiab]) AND (smoke inhalation injury[mesh] OR burns, inhalation[mesh] OR smoke[tiab] OR burn[tiab] OR acute lung injury[mesh] OR critical illness[mesh] OR "respiratory distress syndrome, adult"[MeSH Terms])

**EMBASE – 40 Results**

(‘aerosol’/exp OR ‘nebulizer’/exp OR ‘vaporizer’/exp OR ‘inhalational drug administration’/exp) AND (‘heparin’/exp OR ‘anticoagulant agent’/exp OR ‘antithrombin’/exp OR ‘anticoagulant therapy’/exp) AND (‘lung burn’/exp OR ‘critical illness’/exp OR ‘acute lung injury’/exp OR ‘acute respiratory distress syndrome’/exp)

**EMBASE – 13 results**

('aerosol'/exp OR 'nebulizer'/exp OR 'vaporizer'/exp OR 'inhalational drug administration'/exp) AND ('heparin'/exp OR 'anticoagulant agent'/exp OR 'antithrombin'/exp OR 'anticoagulant therapy'/exp) AND ('lung burn'/exp)

**SCOPUS – 88 results**

(ABS(nebulize OR nebulized OR nebulizer OR nebulizers OR nebulization OR aerosol OR aerosols OR aerosolized OR inhale OR inhaled OR inhalation OR inhalable OR vaporize OR vaporizer OR vaporizers) AND ABS(heparin OR anticoagulant OR anticoagulants OR anticoagulation OR antithrombin OR antithrombins) AND ABS(smoke)

**SCOPUS – 90 results**

(TITLE(nebulize OR nebulized OR nebulizer OR nebulizers OR nebulization OR aerosol OR aerosols OR aerosolized OR inhale OR inhaled OR inhalation OR inhalable OR vaporize OR vaporizer OR vaporizers) AND ABS(heparin OR anticoagulant OR anticoagulants OR anticoagulation OR antithrombin OR antithrombins) AND ABS(smoke OR ventilation)

**SCOPUS – 49 results**

(ABS(nebulize OR nebulized OR nebulizer OR nebulizers OR nebulization OR aerosol OR aerosols OR aerosolized OR inhale OR inhaled OR inhalation OR inhalable OR vaporize OR vaporizer OR vaporizers) AND TITLE(heparin OR anticoagulant OR anticoagulants OR anticoagulation OR antithrombin OR antithrombins) AND ABS(smoke OR ventilation)

**WEB OF SCIENCE – 41 results**

Title=(nebulize OR nebulized OR nebulizer OR nebulizers OR nebulization OR aerosol OR aerosols OR aerosolized OR inhale OR inhaled OR inhalation OR inhalable OR vaporize OR vaporizer OR vaporizers) AND Title=(heparin OR anticoagulant OR anticoagulants OR anticoagulation OR antithrombin OR antithrombins) AND Title=(smoke)

**WEB OF SCIENCE – 13 results**

Title=(nebulize OR nebulized OR nebulizer OR nebulizers OR nebulization OR aerosol OR aerosols OR aerosolized OR inhale OR inhaled OR inhalation OR inhalable OR vaporize OR vaporizer OR vaporizers) AND Title=(heparin OR anticoagulant OR anticoagulants OR anticoagulation OR antithrombin OR antithrombins) AND Topic=(ventilation)

Total 421 🡪 duplicates (205) = total: 216

**APPENDIX 2: Figures – Ventilation data and subgroup analysis**

**Figure S1 –** Kaplan-Meier Estimates of the Probability of the Secondary Outcome. Data for the Kaplan-Meier survival in patients treated with nebulized heparin (black dotted line), and controls (black solid line). Data were censored at 100 days after inclusion. A) Overall cohort, B) Matched cohort.


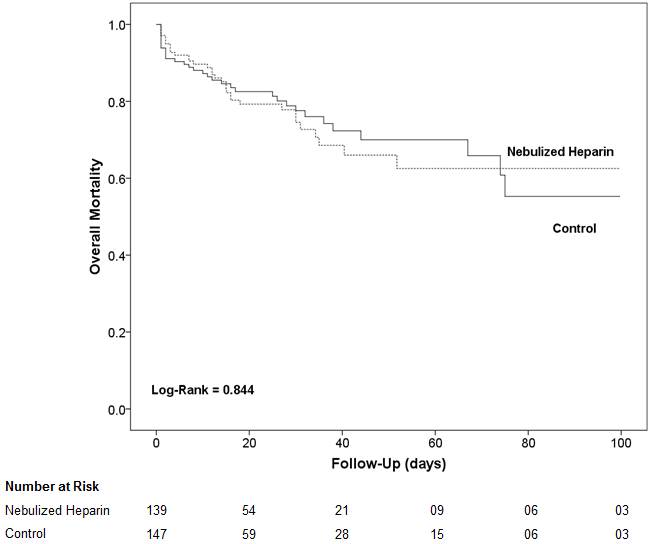

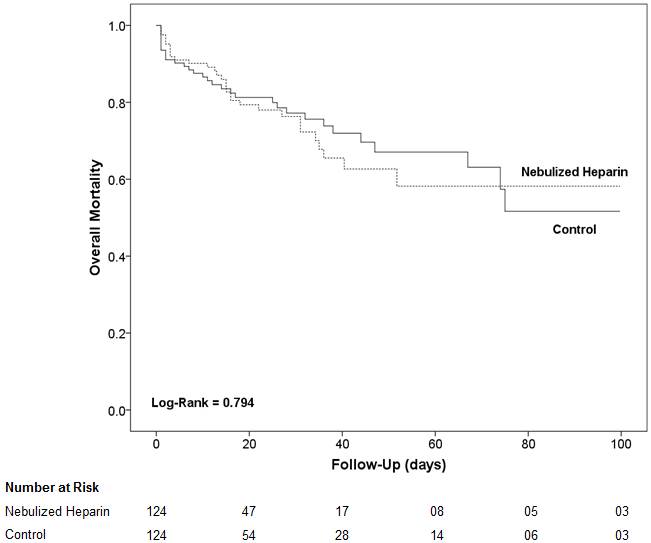


Figure 2B

Figure S1A Figure S1B

**Figure S2 –** Ventilatory parameters in patients treated with nebulized heparin (*black dotted line*) and controls (*black solid line*).

Overall cohort. A, tidal volume; B, peak inspiratory pressure; C, PEEP. *Bars are median and 95% confidence interval*


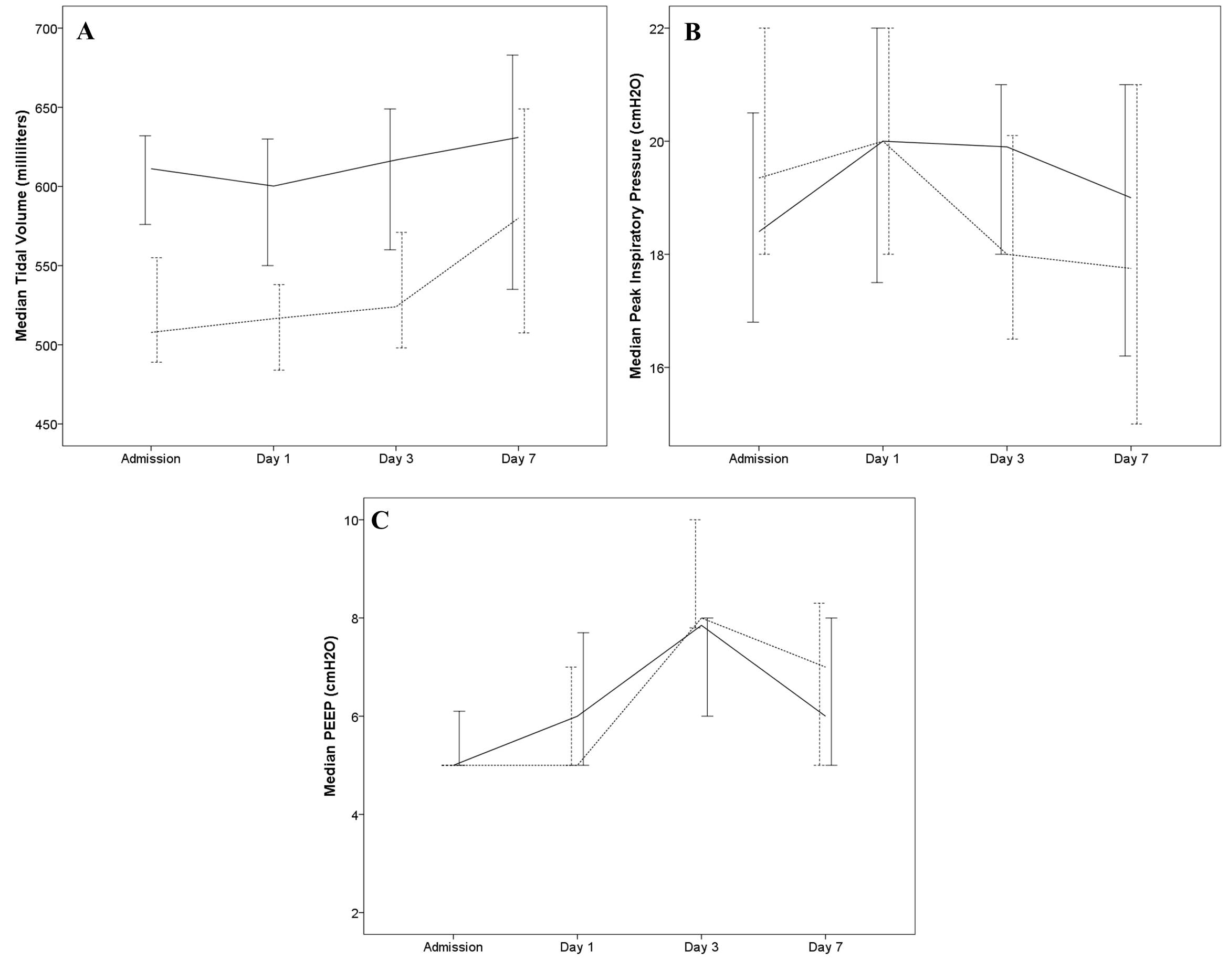


**Figure S3 –** Oxygenation parameters in patients treated with nebulized heparin (*black dotted line*) and controls (*black solid line*). Overall cohort. A, lung injury score; B, PaO_2_ / FiO_2_; C, PaCO_2_; D, pH. *Bars are median and 95% confidence interval*


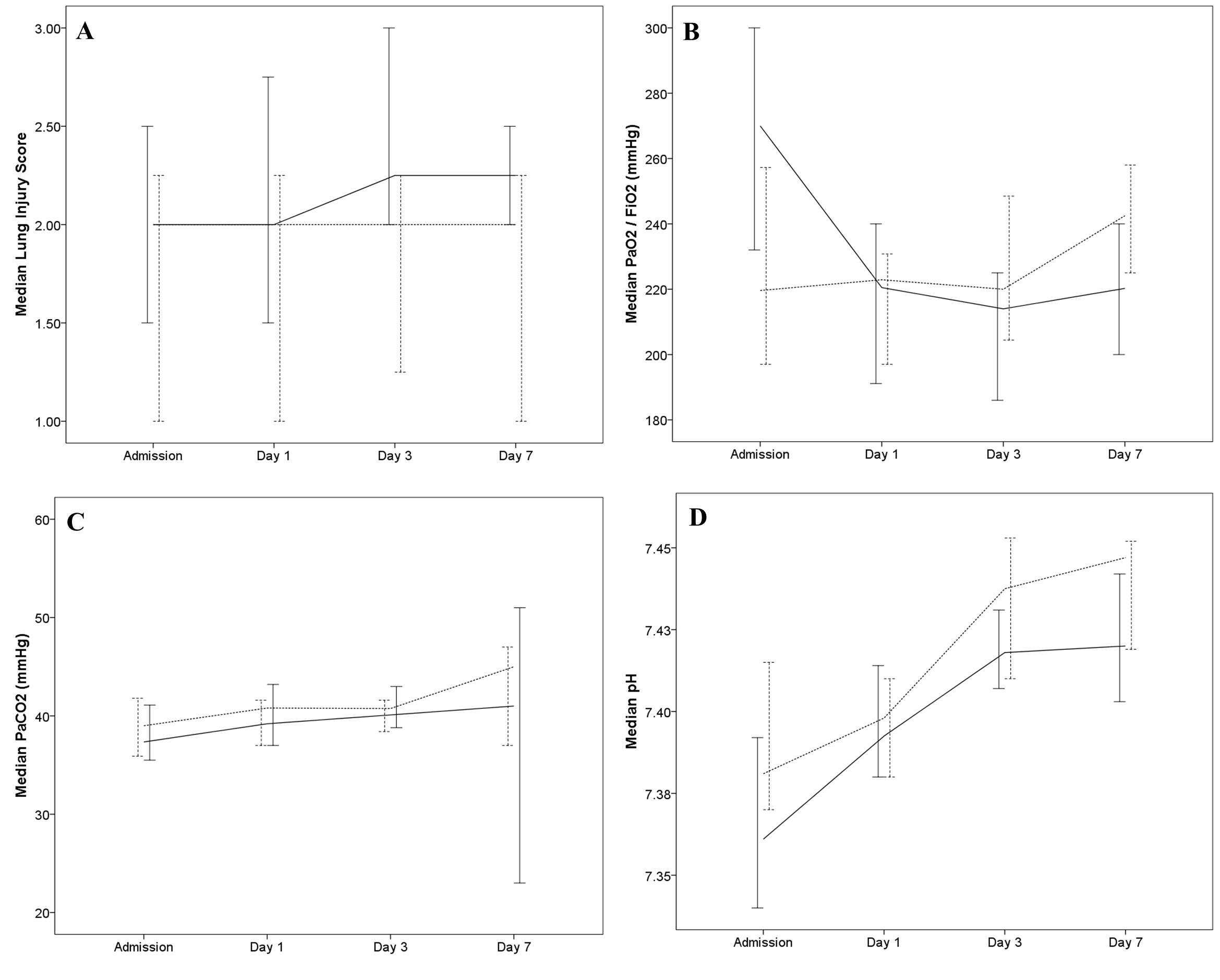


| **Table S1 –** Ventilation and oxygenation parameters | | | | | | | | | |
| --- | --- | --- | --- | --- | --- | --- | --- | --- | --- |
| **Parameters** | **Admission** | | | **Day 01** | | | **Day 03** | | |
|  | **NBZ-Heparin** | **Control** | ***p*** | **NBZ-Heparin** | **Control** | ***p*** | **NBZ-Heparin** | **Control** | ***p*** |
| Tidal volume (milliliters) | 507.8  (436.7 – 614.9)  (N=86) | 611.2  (500.5 – 693.0)  (N=105) | < 0.01 | 516.5  (428.8 – 618.2)  (N=82) | 600.2  (501.5 – 663.2)  (N=86) | 0.001 | 524.0  (430.5 – 655.0)  (N=61) | 616.7  (518.5 – 722.0)  (N=74) | 0.003 |
| Peak pressure (cmH_2_O) | 19.3  (15.2 – 20.0)  (N=102) | 18.4  (14.3 – 25.0)  (N=104) | 0.363 | 20.0  (14.0 – 26.0)  (N=97) | 20.0  (15.0 – 24.0)  (N=87) | 0.829 | 18.8  (15.0 – 24.0)  (N=73) | 19.9  (15.0 – 26.0)  (N=74) | 0.229 |
| PEEP (cmH_2_O) | 5.0  (5.0 – 8.0)  (N=117) | 5.0  (5.0 – 7.6)  (N=118) | 0.078 | 6.0  (5.0 – 9.9)  (N=112) | 5.0  (5.0 – 8.9)  (N=100) | 0.551 | 7.4  (5.0 – 9.8)  (N=88) | 8.0  (5.0 – 10.0)  (N=88) | 0.072 |
| Lung injury score, unit | 2.0  (0.7 – 2.5)  (N=41) | 2.0  (1.2 – 3.0)  (N=39) | 0.288 | 2.0  (0.7 – 2.5)  (N=41) | 2.0  (1.5 – 3.0)  (N=39) | 0.096 | 2.0  (1.0 – 2.5)  (N=41) | 2.2  (1.7 – 3.0)  (N=39) | 0.028 |
| PaO_2_ / FiO_2_, mmHg | 219.6  (158.2 – 316.5)  (N=136) | 270.0  (163.5 – 366.5)  (N=141) | 0.085 | 222.9  (169.9 – 293.7)  (N=112) | 220.5  (161.9 – 295.2)  (N=106) | 0.929 | 215.5  (163.3 – 300.0)  (N=91) | 214.0  (161.3 – 250.7)  (N=89) | 0.204 |
| pH, unit | 7.38  (7.30 – 7.45)  (N=121) | 7.36  (7.29 – 7.44)  (N=128) | 0.330 | 7.39  (7.35 – 7.43)  (N=96) | 7.39  (7.36 – 7.43)  (N=92) | 0.742 | 7.42  (7.37 – 7.46)  (N=76) | 7.42  (7.38 – 7.47)  (N=75) | 0.501 |
| PaCO_2_, mmHg | 39.0  (32.0 – 44.7)  (N=121) | 37.3  (31.7 – 46.5)  (N=128) | 0.818 | 40.8  (33.9 – 45.0)  (N=96) | 39.2  (34.0 – 45.7)  (N=92) | 0.829 | 40.7  (35.7 – 46.4)  (N=76) | 40.1  (35.1 – 44.4)  (N=75) | 0.675 |
| Overall cohort. Values are median (IQR). Not all requested data was available for each study. *NBZ: nebulized; PEEP: positive-end expiratory pressure; N: number of patients.* | | | | | | | | | |

**APPENDIX 3: Tables – Ventilation data and subgroup analysis**

| **Table S2 –** Outcomes of patients in different subgroups (nebulized heparin *vs.* control)^a^ | | | | | | | | |
| --- | --- | --- | --- | --- | --- | --- | --- | --- |
|  | **Age** | | **Population** | | **Dose of Heparin** | | **Intervention** | |
|  | **< 18 years** | **≥ 18 years** | **Burn** | **No-Burn** | **Low^*^** | **High^**^** | **Alone^¶^** | **Combined^¶¶^** |
| Ventilator-Free days  at day 28 | 0.118  (N=23) | 0.297  (N=260) | 0.742  (N=217) | 0.220  (N=66) | 0.999  (N=187) | 0.218  (N=96) | 0.220  (N=66) | 0.742  (N=217) |
| Overall Mortality | 0.87  (0.75 – 1.45)  (N=23) | 0.92  (0.45 – 1.87)  (N=263) | 0.87  (0.44 – 1.74)  (N=220) | 1.23  (0.35 – 4.37)  (N=66) | 0.90  (0.37 – 2.19)  (N=190) | 0.14  (0.01 – 1.77)  (N=96) | 1.23  (0.35 – 4.37)  (N=66) | 0.73  (0.33 – 1.64)  (N=220) |
| Hospital-Free days  at day 28 | 0.169  (N=23) | 0.461  (N=263) | 0.948  (N=220) | 0.688  (N=66) | 0.971  (N=190) | 0.543  (N=96) | 0.688  (N=66) | 0.948  (N=220) |
| Mann-Whitney test for VFD-28 and HFD-28 and logistic regression (OR 95% CI) for mortality. Not all requested data was available for each study. *N: number of patients*  ^a^ adjusted by: age, and baseline PaO_2_ / FiO_2_  * low defined as 30.000 units/day  ** high defined as 60.000 to 150.000 units/day  ¶ only heparin  ¶¶ heparin plus bronchodilators and/or mucolytics | | | | | | | | |

**Appendix 4: Propensity Matched Cohort**

Propensity scores were estimated for each patient with logistic regression using two clinically relevant baseline characteristics (age, and baseline PaO_2_/FiO_2_). The propensity score is the propensity from 0 to 1 to be in the group receiving nebulized anticoagulants given a set of know variables and is used to attempt to adjust for potential selection bias, confounding, and differences between the groups in observational studies. Each individual received the propensity score that incorporated all non–missing variables for that individual. Accordingly, we constructed a propensity score–matched cohort. Matching was performed using nearest neighbor matching without replacement, with each patient receiving nebulized heparin matched to one patient not receiving nebulized heparin (matching 1:1). A caliper width of 0.15 of the standard deviation of the logit of the propensity score was used for the development of matching. Absolute standardized differences were computed to evaluate matching effectiveness; values less than 10% and closer to zero demonstrate a more balanced cohort.

| **Table S3–** Characteristics of the patients included in the individual patient data analysis | | | |
| --- | --- | --- | --- |
| **Variables** | **Nebulized Heparin**  **(N= 124)** | **Control**  **(N = 124)** | **SD (%), *p*** |
| Age, years | 50.5 (34.2 – 68.7)  (N=124) | 46.5 (35.0 – 66.0)  (N=124) | 1.9, 0.64 |
| Gender, male (%) | 81 (65.3) | 88 (71.0) | -13.2, 0.34 |
| APACHE III | 22.0 (17.0 – 30.0)  (N=51) | 24.0 (15.0 – 32.0)  (N=39) | -0.8, 0.92 |
| % TBSA | 25.0 (13.0 – 51.5)  (N=79) | 33.0 (19.0 – 51.9)  (N=88) | -9.6, 0.30 |
| Dosage of heparin, units/day | 30,000  (30,000 – 52,500) | 0.0  (0.0 – 0.0) | --- |
| Dosage of NAC, mg/day | 3,600 (3,600 – 3,600) | 0.0 (0.0 – 0.0) | --- |
| Duration of treatment | 7.0 (2.0 – 11.0) | 0.0 (0.0 – 0.0) | --- |
| Baseline LIS | 2.0 (0.7 – 2.5)  (N=35) | 2.0 (1.2 – 3.0)  (N=39) | -19.0, 0.45 |
| Baseline PaO_2_ / FiO_2_ | 219.6  (158.6 – 312.5)  (N=124) | 256.5  (156.5 – 344.5)  (N=124) | -10.1, 0.54 |
| Matched cohort. Values are median (IQR) or no./total no. (%). Not all requested data was available for each study.  *SD: standardized difference; TBSA: total burn surface area; NAC: N-acetylcysteine; LIS: lung injury score; N: number of patients* | | | |

| **Table S4–** Primary and secondary outcomes^ǁ^ | | | | |
| --- | --- | --- | --- | --- |
| **Variables** | **Nebulized Heparin**  **(N=124)** | **Control**  **(N=124)** | **Odds Ratio**  **(95% CI)** | ***p*** |
| ***Primary outcome*** |  |  |  |  |
| Ventilator-Free days at day 28 | 15.5 (0.0 – 23.0)  (N=124) | 4.5 (0.0 – 20.0)  (N=122) |  | 0.133 |
| ***Secondary outcomes*** |  |  |  |  |
| Overall Mortality | 32/124 (25.8)  (N=124) | 33/124 (26.6)  (N=124) | 0.96 (0.54 – 1.69) | 0.885 |
| PaO_2_ / FiO_2_ at day seven,  mmHg | 241.0  (203.0 – 281.9)  (N=60) | 220.2  (177.1 – 292.3)  (N=82) |  | 0.202 |
| LIS at day seven | 2.1 (1.1 – 2.7)  (N=52) | 2.2 (1.7 – 3.0)  (N=56) |  | 0.156 |
| ICU-Free days at day 28 | 13.7 (0.0 – 19.7)  (N=51) | 8.0 (0.0 – 19.0)  (N=39) |  | 0.374 |
| Hospital-Free days at day 28 | 1. (0.0 – 12.0)   (N=124) | 0.0 (0.0 – 12.5)  (N=124) |  | 0.285 |
| ǁ values are median (IQR) and others are no./total no. (%). *** Adjusted by: age and baseline PaO_2_ / FiO_2_.  ** presented as hazard ratio adjusted by: age, %TBSA, and baseline PaO_2_ / FiO_2_*.*  Not all requested data was available for each study. *LIS: lung injury score; CI: confidence interval; N: number of patients* | | | | |

| **Table S5 –** Ventilation and oxygenation parameters | | | | | | | | | |
| --- | --- | --- | --- | --- | --- | --- | --- | --- | --- |
| **Parameters** | **Admission** | | | **Day 01** | | | **Day 03** | | |
|  | **NBZ-Heparin** | **Control** | ***p*** | **NBZ-Heparin** | **Control** | ***p*** | **NBZ-Heparin** | **Control** | ***p*** |
| Tidal volume (milliliters) | 505.0  (421.0 – 604.0)  (N=75) | 607.0  (487.0 – 696.0)  (N=87) | < 0.001 | 512.0  (421.2 – 591.5)  (N=89) | 560.0  (498.0 – 661.0)  (N=87) | < 0.001 | 512.0  (420.0 – 645.0)  (N=63) | 593.0  (460.0 – 700.0)  (N=78) | 0.028 |
| Peak pressure (cmH_2_O) | 19.8  (15.2 – 26.0)  (N=91) | 20.0  (15.0 – 25.0)  (N=86) | 0.669 | 22.0  (16.0 – 28.0)  (N=104) | 22.0  (16.1 – 27.5)  (N=89) | 0.828 | 20.1  (15.0 – 26.0)  (N=75) | 21.0  (17.9 – 28.0)  (N=78) | 0.209 |
| PEEP (cmH_2_O) | 5.0  (5.0 – 8.0)  (N=103) | 5.0  (5.0 – 8.0)  (N=100) | 0.189 | 7.0  (5.0 – 10.0)  (N=116) | 6.5  (5.0 – 10.0)  (N=101) | 0.903 | 8.0  (5.0 – 10.0)  (N=87) | 9.3  (5.0 – 12.0)  (N=92) | 0.095 |
| Lung injury score, unit | 2.0  (0.7 – 2.5)  (N=35) | 2.0  (1.2 – 3.0)  (N=39) | 0.447 | 2.0  (1.2 – 2.7)  (N=54) | 2.0  (1.5 – 3.0)  (N=57) | 0.393 | 2.1  (1.5 – 2.7)  (N=54) | 2.2  (1.7 – 3.0)  (N=57) | 0.187 |
| PaO_2_ / FiO_2_, mmHg | 219.6  (158.6 – 312.5)  (N=124) | 256.5  (156.5 – 344.5)  (N=124) | 0.544 | 200.0  (149.4 – 275.5)  (N=119) | 215.7  (162.2 – 252.8)  (N=108) | 0.674 | 215.6  (161.7 – 283.8)  (N=95) | 215.7  (162.2 – 252.8)  (N=96) | 0.484 |
| pH, unit | 7.38  (7.30 – 7.44)  (N=111) | 7.36  (7.29 – 7.44)  (N=110) | 0.293 | 7.39  (7.36 – 7.43)  (N=106) | 7.39  (7.36 – 7.44)  (N=94) | 0.779 | 7.44  (7.39 – 7.47)  (N=82) | 7.41  (7.37 – 7.46)  (N=80) | 0.108 |
| PaCO_2_, mmHg | 39.8  (32.5 – 44.9)  (N=111) | 38.5  (32.1 – 47.0)  (N=110) | 0.645 | 42.5  (34.5 – 46.0)  (N=106) | 39.7  (34.0 – 46.0)  (N=94) | 0.375 | 41.5  (36.7 – 47.0)  (N=82) | 41.1  (37.0 – 47.0)  (N=80) | 0.883 |
| Values are median (IQR). Not all requested data was available for each study. *NBZ: nebulized; PEEP: positive-end expiratory pressure; N: number of patients.* | | | | | | | | | |

| **Table S6 –** Post-hoc sensitivity analysis in the matched cohort (control vs heparin) | | | |
| --- | --- | --- | --- |
|  |  | **Ventilator-Free days at day 28** | **Hospital-Free days at day 28** |
| **Age** | **< 18 years** | 24.0 (14.0 – 27.0) vs. 16.0 (0.0 – 23.0)  *p* = 0.126  (N=18) | 14.0 (0.0 – 19.0) vs. 0.0 (0.0 – 10.0)  *p* = 0.246  (N=18) |
|  | **≥ 18 years** | 3.0 (0.0 – 20.0) vs. 15.0 (0.0 – 23.0)  *p* = 0.056  (N=228) | (0.0 – 9.5) vs. 0.0 (0.0 – 12.0)  *p* = 0.164  (N=230) |
| **Population** | **Burn** | 0.0 (0.0 – 16.5) vs. 12.0 (0.0 – 22.4)  *p* = 0.070  (N=164) | (0.0 – 6.0) vs. 0.0 (0.0 – 10.0)  *p* = 0.119  (N=164) |
|  | **Non-Burn** | 19.0 (0.0 – 22.0) vs. 18.0 (0.0 – 25.5)  *p* = 0.727  (N=60) | 3.0 (0.0 – 17.0) vs. 3.0 (0.0 – 15.5)  *p* = 0.860  (N=60) |
| **Heparin dose** | **Low^*^** | 6.5 (0.0 – 21.7) vs. 16.0 (0.0 – 25.0)  *p* = 0.229  (N=203) | (0.0 – 14.2) vs. 0.0 (0.0 – 14.0)  *p* = 0.274  (N=205) |
|  | **High^**^** | 0.0 (0.0 – 3.5) vs. 13.0 (0.0 – 22.2)  *p* = 0.007  (N=43) | (0.0 – 0.0) vs. 0.0 (0.0 – 6.9)  *p* = 0.224  (N=43) |
| **Tidal Volume** | **≤ 560** | 14.0 (0.0 – 20.0) vs. 17.0 (0.0 – 24.2)  *p* = 0.289  (N=81) | (0.0 – 10.0) vs. 2.5 (0.0 – 15.5)  *p* = 0.330  (N=81) |
|  | **> 560** | 4.5 (0.0 – 24.7) vs. 0.0 (0.0 – 23.5);  *p* = 0.626  (N=81) | (0.0 – 15.6) vs. 0.0 (0.0 – 12.0)  *p* = 0.575  (N=81) |

Values are medians (IQR). Not all requested data was available for each study.

* low defined as 30.000 units/day

** high defined as 60.000 to 150.000 units/day

**Appendix 5: Assessment of Quality of Evidence of included studies**

1. Randomized Study: Cochrane Collaboration’s tool for assessing risk of bias

| Author (year) |  | Random sequence generation | Allocation concealment | Blinding | Blinding of outcome assessment | Incomplete outcome data | Selective reporting | Other Bias | Reference |
| --- | --- | --- | --- | --- | --- | --- | --- | --- | --- |
| Dixon  (2010) | Part 1  Assessment | Block randomization in random blocks of two to eight | Sequentially numbered, opaque, sealed envelopes | Double blinding: participants and key study personnel; unlikely that the blinding could have been broken | Blinding of outcome assessment ensured, and unlikely that the blinding could have been broken | No missing outcome data; all patients were included in the final analysis | Study registered at a clinical trial register | Unlikely | [40] |
|  | Part 2  Judgement | Low Risk | Low Risk | Low Risk | Low Risk | Low Risk | Low Risk | Low Risk |  |

**Legend.** Each domain in the tool includes one or more specific entries in a ‘Risk of bias’ table. The first part of the tool describes what was reported to have happened in the study, in sufficient detail to support a judgement about the risk of bias. The second part of the tool assigns a judgement relating to the risk of bias for that entry. This is achieved by assigning a judgement of ‘Low risk’ of bias, ‘High risk’ of bias, or ‘Unclear risk’ of bias.

1. **Nonrandomized studies – Newcastle-Ottawa Quality Assessment Scale**

(<http://www.ohri.ca/programs/clinical_epidemiology/oxford.asp> )

| **Author (year)** | **Quality assessment** | | | **Reference** |
| --- | --- | --- | --- | --- |
|  | 1. Selection | 1. Comparability | 1. Outcome |  |
| Holt ^a^  (2008) |  |  |  | [51] |
| Dixon ^b^  (2008) |  | - |  | [39] |
| Miller ^c^  (2009) |  |  |  | [53] |
| Kashefi ^d^  (2014) |  |  |  | [52] |

**Legend.** ‘High’ quality choices are identified with a star ( ).

Maximum number of starts per item: Selection (4 stars), Comparability (2 stars), Outcome (3 stars).

**Selection**

**^1a^** representative cohort of mechanically ventilated patients with inhalation injury; inclusion of consecutive patients over a defined period of time; Historical control patients from the same hospital; Allocation: lack of allocation concealment, choice whether to start treatment with nebulized heparin or not was left at the discretion of attending physicians; Ascertainment of exposure: orders from medical or pharmacy records (start of the protocol), no information on the number of scheduled doses that were withheld. Outcome of interest not present at the start of the study

**^1b^** representative cohort for mechanically ventilated patients with acute lung injury; Allocation: not described; no controls; Ascertainment of exposure: all patients were treated with heparin nebulizations (dose escalating); last dose at 36 hours for all patients except for the 4000.00U/day group in which it was administered at 42 hours. Outcome of interest not present at the start of the study

**^1c^** representative cohort of mechanically ventilated patients with inhalation injury; inclusion of consecutive patients over a defined period of time; Historical control patients from the same hospital; Allocation: lack of allocation concealment; Ascertainment of exposure: all patients in the experimental group were treated with heparin nebulizations; no information on the number of scheduled doses that were withheld. Outcome of interest not present at the start of the study: ‘Upon study entry there were no differences in LIS between groups.’

**^1d^** representative cohort of mechanically ventilated patients with inhalation injury; inclusion of consecutive patients over a defined period of time; Historical control patients from the same hospital; Allocation: lack of allocation concealment; Ascertainment of exposure: all patients in the experimental group were treated with heparin nebulizations; no information on the number of scheduled doses that were withheld. Outcome of interest not present at the start of the study;

**Comparability**

**^2a^** historical controls; similar demographics (such as age and severity of injury) between treatment and controls

**^2b^** no control group, prospective open label phase I study

**^2c^** historical controls; study patients were matched to patient in the treatment group based on their admission APACHE-III scores

**^2d^** historical controls; controls were matched 1:1 for sex, burn severity and age within the decade.

**Outcome**

**^3a^** outcome variables were gathered from the ABA/TRACS database and chart review; No information on length of follow-up however data on outcomes such as length of stay and mortality were provided. Not stated whether this was ICU- of hospital length of stay and mortality.

**^3b^** open label study. Lung function and anticoagulant effects assessed at predefined fixed time points from baseline up to several hours after the last nebulization. No data on length of follow-up however, data on hospital- and ICU length-of-stay and hospital mortality were provided.

**^3c^** no description on the assessment of outcome; No data on length of follow-up however patients remained in the study until death or patient recovery and discharge (survival curve provided data up to 175 days).

**^3d^** outcome variables were acquired from the institutional Trauma Registry. No data on length of follow-up however data on length of stay was provided. Not stated whether this was ICU- of hospital length of stay.
